# Epidural Block in a Patient With Melkersson-Rosenthal Syndrome: A Case Report

**DOI:** 10.7759/cureus.61282

**Published:** 2024-05-29

**Authors:** Acácia Silva, Mariana F Lima, Carlos Barbosa, Helder Cardoso

**Affiliations:** 1 Anesthesiology, Unidade Local de Saúde Tâmega e Sousa, Penafiel, PRT

**Keywords:** anesthesia, rare genetic diseases, melkerson-rosenthal syndrome, difficult airway, labor analgesia

## Abstract

This case report describes the first known application of an epidural block for labor analgesia in a patient with Melkersson-Rosenthal syndrome (MRS), a rare disorder that may present sudden and threatening airway complications. A tailored epidural protocol effectively mitigated symptom exacerbation, facilitating a complication-free vaginal delivery. This report not only enriches the sparse literature on anesthesia in patients with MRS but also provides a crucial review of perioperative considerations for administering either general or regional anesthesia in similar cases.

## Introduction

Melkersson-Rosenthal syndrome (MRS) is a rare condition with an annual incidence of 80 per 100,000 people, most commonly occurring between the ages of 20 and 30 [1]. It is characterized by recurrent episodes of facial edema, peripheral facial paralysis, and a fissured tongue. The edema is more frequent in the upper lip but can manifest in other areas, such as the tongue, oropharynx, and larynx [2]. The fissured tongue may lead to permanent tongue hypertrophy and fibrosis. Other relevant atypical symptoms include involvement of other cranial nerves, meningeal signs, and electrocardiographic alterations [2]. The etiology of MRS is unknown, but genetic factors, chronic infections, allergies, and a dysregulated autoimmune system appear to contribute [3]. There is no specific treatment for MRS. Systemic corticosteroids are frequently used to induce remission, and refractory cases may benefit from immunosuppressants such as methotrexate or anti-Tumor necrosis factor (TNF) agents [1,4].

There are few published case reports on the anesthetic management of patients with MRS. To the best of our knowledge, this is the first case report of labor analgesia in a patient with a confirmed MRS diagnosis. We will review clinical manifestations essential for understanding and managing MRS in overall anesthetic scenarios.

## Case presentation

A 28-year-old first-time mother at full term was admitted for labor induction. Her medical history was relevant for gestational diabetes, allergic rhinitis, and MRS. Diagnosed nine years ago through a lip biopsy following recurrent episodes of upper lip and tongue edema and a permanent fissured tongue, she was treated with oral deflazacort (0.5 mg/kg per day), achieving remission after two years. Since then, mild MRS exacerbations have been effectively managed with topical corticosteroids. Cocoa ingestion was identified as a trigger for her facial edema; however, no pharmacological triggers were identified. She had no known allergies.

**Figure 1:**
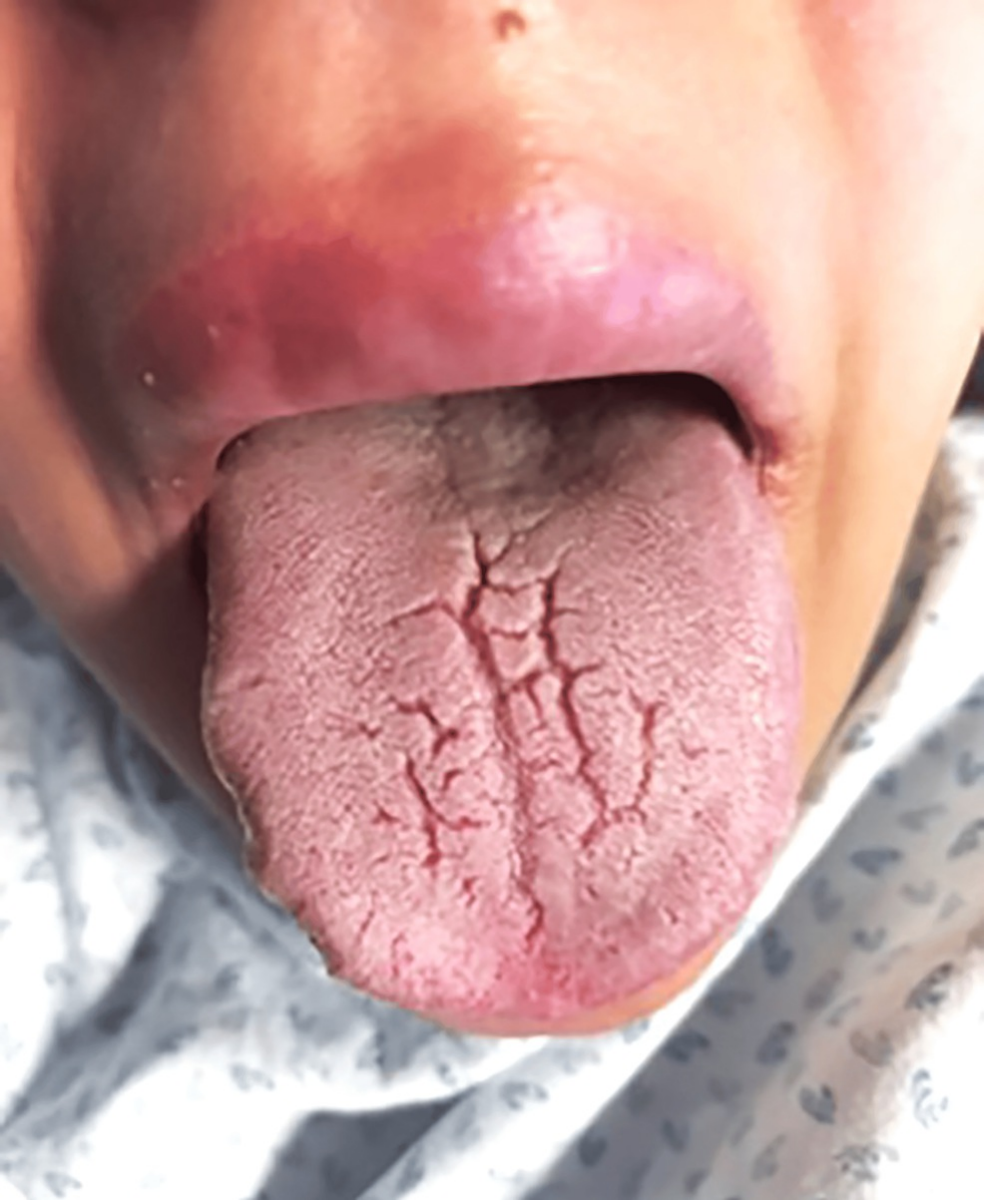
Melkersson-Rosenthal syndrome features present in the patient: upper lip edema and fissured tongue.

During the evaluation, she exhibited mild upper lip edema and a fissured but normally-sized tongue (Figure 1).

No signs or symptoms of oropharyngeal or laryngeal edema were noticed. She was classified as Mallampati II, and no indicators of a difficult airway were observed.

Labor epidural analgesia was conducted after patient consent and standard American Society of Anesthesiologists (ASA) monitoring. An epidural catheter was positioned at L3-L4 level, and a fractionated bolus of 10 mL of 0.2% ropivacaine was given, followed by a labor analgesia protocol of 10 mL of 0.2% ropivacaine hourly.

The patient remained hemodynamically stable, with adequate pain control and no signs or symptoms of MRS acute exacerbations during care. Vaginal delivery occurred without complications.

## Discussion

MRS, although a rare and predominantly benign condition, presents significant anesthetic challenges in patient care. As previously noted, a key characteristic of MRS is orofacial involvement, notably tongue hypertrophy and edema affecting the lips, tongue, oropharynx, and larynx. Crucially, the incomplete resolution of this edema can result in fibrosis and morphological distortion of these tissues [4]. A notable case from 1993 exemplifies these challenges: a young woman with undiagnosed MRS experienced respiratory obstruction, necessitating tracheal intubation. This procedure was complicated, requiring the insertion of a bougie through a significantly narrowed glottic lumen [5].

In managing MRS, it is imperative to conduct a comprehensive airway examination and prepare for potential difficulties. Equally important is a detailed review of the patient's clinical history. This review should focus on previous episodes of exacerbation, the anatomical structures affected, factors triggering edema, any known drug allergies, and the occurrence of side effects related to corticosteroids or other immunosuppressive therapies.

For patients with MRS, prioritizing locoregional anesthesia is advisable when feasible [6]. Additionally, it is important to avoid drugs known for inducing histamine release, such as atracurium, mivacurium, morphine, and meperidine [7]. This caution is because up to 30% of patients may exhibit clinical signs of histamine release during anesthesia. Consequently, the prophylactic use of corticosteroids and anti-histamine drugs can be considered. Moreover, given that both cold and warm temperatures can trigger urticaria and angioedema [8], maintaining normothermia throughout the perioperative period is crucial for these patients [6].

Throughout the perioperative period, it is essential to continue specific treatment for MRS to reduce the risk of exacerbations. Additionally, patients who have recently received corticosteroid therapy may require intraoperative supplementation to prevent acute suprarenal insufficiency [9]. Furthermore, patients currently undergoing immunosuppressant therapy should be regarded as having an increased risk for infectious complications [10].

At presentation, patients may display atypical symptoms, such as changes on an electrocardiogram (ECG), making it crucial to perform a preoperative ECG to identify any baseline abnormalities. These findings may lead to further investigations, if required. Additionally, if neurological symptoms, such as meningeal signs or cranial nerve involvement, are present, a comprehensive evaluation is essential to exclude other possible causes and document these changes prior to any anesthetic intervention. Table 1 provides essential information for anesthesiologists managing patients with MRS.

**Table 1:** Summary of perioperative considerations for managing patients with MRS

Quick review of Melkersson-Rosenthal syndrome
MRS is a rare condition of unknown etiology.
It is characterized by episodes of edema, potentially affecting the tongue, oropharynx, and larynx.
MRS can present significant airway challenges due to edema and tissue distortion.
To avoid exacerbations, it is crucial to maintain regular medication, including the consideration of prophylactic use of corticosteroids and antihistamines to minimize mucosal edema.
Patients with MRS may be under treatment with corticosteroids and immunosuppressants. Corticosteroid supplementation should be considered to avoid suprarenal suppression and heightened infectious risk should be managed accordingly.
It is advisable to avoid known triggers and histamine-releasing drugs such as atracurium, mivacurium, morphine, and meperidine.
Maintaining normal body temperature is important to decrease the risk of angioedema.
Airway manipulation should be minimized and locoregional anesthesia should be chosen whenever possible.
Ambulatory surgery may not be suitable for patients with MRS.

In discussing our case, it is noteworthy that the patient exhibited no signs of a difficult airway. However, labor inherently increases the risk of airway complications, primarily due to factors such as mucosal edema, an elevated risk of gastric aspiration, and changes in respiratory physiology. Epidural analgesia was effectively used to manage pain during vaginal delivery and could have also been employed as the anesthetic method for an urgent cesarean section if needed. Nevertheless, the possibility of requiring general anesthesia and airway instrumentation remained, especially in the event of an emergent cesarean. In such a scenario, employing rapid sequence induction with a video laryngoscope for intubation, alongside the use of a narrow tracheal tube equipped with a pre-loaded stylet, would be optimal to increase the likelihood of success on the first attempt while minimizing the risk of exacerbating mucosal edema.

## Conclusions

While the choice of loco-regional anesthesia is clear and straightforward for any anesthesiologist managing potential airway difficulties, this case adds valuable insights to the sparse literature on MRS during the peripartum period. The administration of 0.2% ropivacaine via an epidural catheter infusion did not worsen the patient's symptoms, showcasing the safety and efficacy of this anesthetic approach.
